# On an Approximate Solution of the Cauchy Problem for Systems of Equations of Elliptic Type of the First Order

**DOI:** 10.3390/e24070968

**Published:** 2022-07-13

**Authors:** Davron Aslonqulovich Juraev, Ali Shokri, Daniela Marian

**Affiliations:** 1 Department of Natural Science Disciplines, Higher Military Aviation School of the Republic of Uzbekistan, Karshi 180117, Uzbekistan; juraevdavron12@gmail.com; 2 Department of Mathematics, Anand International College of Engineering, Jaipur 303012, Rajasthan, India; 3 Department of Mathematics, Faculty of Basic Sciences, University of Maragheh, Maragheh 83111-55181, Iran; shokri@maragheh.ac.ir; 4 Department of Mathematics, Technical University of Cluj-Napoca, 28 Memorandumului Street, 400114 Cluj-Napoca, Romania

**Keywords:** integral formula, regularization of the Cauchy problem, approximate solution, Carleman matrix, family of vector functions, Bessel and Hankel functions, 35J46, 35J56

## Abstract

In this paper, on the basis of the Carleman matrix, we explicitly construct a regularized solution of the Cauchy problem for the matrix factorization of Helmholtz’s equation in an unbounded two-dimensional domain. The focus of this paper is on regularization formulas for solutions to the Cauchy problem. The question of the existence of a solution to the problem is not considered—it is assumed a priori. At the same time, it should be noted that any regularization formula leads to an approximate solution of the Cauchy problem for all data, even if there is no solution in the usual classical sense. Moreover, for explicit regularization formulas, one can indicate in what sense the approximate solution turns out to be optimal.

## 1. Introduction

Most of actively developing modern area of scientific knowledge is the theory of correctly and incorrectly posed problems, most of which have practical value and require decision making in uncertain or contradictory conditions. The development and justification of methods for solving such a complex problems as ill-posed ones is intensely investigated of the present time. The results regarding ill-posed problems are a scientific research apparatus for many scientific areas, such as differentiation of approximately given functions, solving inverse boundary value problems, solving problems of linear programming and control systems, solving systems of linear equations, degenerate or ill-conditioned, etc.

The concept of a “well-posed problem” was first introduced by the French mathematician J. Hadamard in 1923 when he considered for partial differential equations of mathematical physics the extension of boundary value problems. The concept of correctness of problems was the basis for the classification of boundary value problems. In this case, the correctness of the problem statement was ensured by the fulfillment of two conditions: the existence of a solution and its uniqueness. The requirement of stability of the solution was subsequently attached to the first two by other mathematicians already during a more in-depth study of this class of problems. Problems in which any of the three conditions for the correct formulation of the problem (stability, existence or uniqueness) is not fulfilled belong to the class of ill-posed problems. The need to solve unstable problems like the one above requires a more precise definition of the solution to the problem (example Hadamard, see, for instance [1], p. 39).

We will say that the problem is correctly posed according to Tikhonov (See [2]) if:

(1) the solution of the problem exists in some class;
(2) the solution is unique in this class;
(3) the solution of the problem depends continuously on the input data.

The Cauchy problem for systems of elliptic equations with constant coefficients belongs to the family of ill-posed problems: the solution of the problem is unique, but unstable. For more details on this subject can be consulted [2,3,4,5,6,7,8,9,10]. The paper studies the construction of exact and approximate solutions to the ill-posed Cauchy problem for matrix factorizations of the Helmholtz equation. Such problems naturally arise in mathematical physics and in various fields of natural science (for example, in electro-geological exploration, in cardiology, in electrodynamics, etc.). In general, the theory of ill-posed problems for elliptic systems of equations has been sufficiently developed thanks to the works of A.N. Tikhonov, V.K. Ivanov, M.M. Lavrent’ev, N.N. Tarkhanov and others famous mathematicians. Among them, the most important for applications are the so-called conditionally well-posed problems, characterized by stability in the presence of additional information about the nature of the problem data. One of the most effective ways to study such problems is to construct regularizing operators. For example, this can be the Carleman-type formulas (as in complex analysis) or iterative processes (the Kozlov-Maz’ya-Fomin algorithm, etc.) [10]. Boundary problems, as well as numerical solutions of some problems, are considered in works [11,12,13,14,15,16,17,18,19,20,21,22,23,24,25,26,27,28,29,30,31,32].

We construct, in this paper, an explicit Carleman matrix, regarding the Cauchy problem for Helmholtz’s equation, based on works [7,8,9,10]. Using this, a regularized solution of the Cauchy problem for the matrix factorization of the Helmholtz equation is given. Some formulas of Carleman type for certain equations and systems of elliptic type are given in [7,8,9,10,33,34,35,36,37,38,39]. In work [33] it was considered the Cauchy problem for the Helmholtz equation in an arbitrary bounded plane domain with Cauchy data, known only on the region boundary. In [40], the Cauchy problem for the Helmholtz equation in a bounded domain was considered. In the present study, we have constructed an approximate solution of the Cauchy problem for matrix factorizations of the Helmholtz equation in a two-dimensional unbounded domain.

In many well-posed problems it is not easy to compute the values of the function on the whole boundary. Thus, one of the important problems in the theory of differential equations is the reconstructing of the solution of systems of equations of first order elliptic type, factorizing the Helmholtz operator (see, for instance [34,35,36,37,38,39]).

The Cauchy problem for elliptic equations was investigated in [6,7,40] and subsequently developed in [9,10,33,35,36,37,38,39].

Next we establish the notations used in the paper.

Let $x=\left(x_{1},x_{2}\right)\in R^{2},\;y=\left(y_{1},y_{2}\right)\in R^{2}.$ We consider in $R^{2}$ an unbounded domain, simply-connected, $\Omega \subset R^{2}$. We suppose that its border ∂Ω is piece wise smooth and is composed of the plane *T*: $y_{2}=0$ and a smooth curve Σ lying in the half-space $y_{2}>0$, that is, ∂Ω=Σ⋃T.

Let:

$$r=\left|y-x\right|,\;\alpha =\left|y_{1}-x_{1}\right|,\;z=i\sqrt{a^{2}+\alpha^{2}}+y_{2},\;a\geq 0,$$

$\partial_{x}={\left(\partial_{x_{1}},\partial_{x_{2}}\right)}^{T},\;\partial_{x}\to \xi^{T},\;\xi^{T}=\left(\begin{array}{c} \xi_{1}\\ \xi_{2} \end{array}\right)$ transposed vector ξ,

$$V\left(x\right)={\left(V_{1}\left(x\right),\ldots ,V_{n}\left(x\right)\right)}^{T},\;v^{0}=\left(1,\ldots ,1\right)\in R^{n},\;n=2^{m},\;m=2,$$

$$E\left(w\right)=∥\begin{array}{cccc} w_{1} & 0 & \ldots & 0\\ 0 & w_{2} & \ldots & 0\\ \ldots & \ldots & \ddots & \ldots \\ 0 & 0 & 0 & w_{n} \end{array}∥-\mathrm{diagonal}\;\mathrm{matrix},w=\left(w_{1},\ldots ,w_{n}\right)\in R^{n}.$$

We consider a (n×n)—dimensional matrix $D(\xi^{T})$ such that

$$D^{*}\left(\xi^{T}\right)D\left(\xi^{T}\right)=E\left(\left({\left|\xi \right|}^{2}+\lambda^{2}\right)v^{0}\right),$$

where $D^{*}\left(\xi^{T}\right)$ is the Hermitian conjugate matrix $D(\xi^{T},)$λ∈R and the elements of $D(\xi^{T})$ are linear functions with constant coefficients of the complex plane.

We also consider the system of differential equations:

(1) $$D\left(\partial_{x}\right)V\left(x\right)=0,\;x\in \Omega ,$$

$D\left(\partial x\right)$ being the matrix of first-order differential operators.

Let $A\left(\Omega \right)=\{V:\bar{\Omega }⟶R^{n}|V$ is continuous on $\bar{\Omega }=\Omega \cup \partial \Omega$ and V satisfies the system (1)}.

## 2. Statement of the Cauchy Problem

Let $f\in C(\Sigma ,R^{n})$. We formulate the following Cauchy problem for the system (1):

Let V(y)∈A(Ω) such that

(2) $${\left.V(y)\right|}_{\Sigma }=f\left(y\right),\;y\in \Sigma .$$

We specify that V(y) is defined on Ω, knowing f(y),y∈Σ.

If V(y)∈A(Ω), then

(3) $$V\left(x\right)=\int_{\partial \Omega }L\left(y,\;x;\lambda \right)V\left(y\right)ds_{y},\;x\in \Omega ,$$

$$L\left(y,\;x;\lambda \right)=\left(E\left(\varphi_{2}\left(\lambda r\right)v^{0}\right)D^{*}\left(\partial_{x}\right)\right)D\left(t^{T}\right),$$

where $t=(t_{1},t_{2})$ means the unit exterior normal at a point y∈∂Ω and $\varphi_{2}\left(\lambda r\right)$ represents the fundamental solution of the Helmholtz equation in $R^{2},$ that is

(4) $$\begin{array}{c} \varphi_{2}\left(\lambda r\right)=-\frac{i}{4}H_{0}^{\left(1\right)}\left(\lambda r\right), \end{array}$$

$H_{0}^{\left(1\right)}\left(\lambda r\right)$ being the the Hankel function of the first kind [41].

An entire function K(z) is introduced, taking real values for real part of *z*, (z=a+ib,a,b∈R) and such that:

(5) $$\begin{array}{c} K\left(z\right)\neq 0,\;\underset{b\geq 1}{\sup}\left|b^{p}K^{\left(p\right)}\left(z\right)\right|=B\left(a,\;p\right)<\infty ,\\ -\infty <a<\infty ,\;p\in \{0,1,2\}. \end{array}$$

Let

(6) Ψ(y,x;λ)=−12πK(x2)∫0∞ImK(z)z−x2aI0(λa)a2+α2da,fory≠x,

where $I_{0}\left(\lambda a\right)=J_{0}\left(i\lambda a\right)$ is the zero order Bessel function of the first kind [4].

We remark that (3) holds if we consider

(7) $$\Psi \left(y,x;\lambda \right)=\varphi_{2}\left(\lambda r\right)+g\left(y,x;\lambda \right),$$

instead $\varphi_{2}\left(\lambda r\right)$, g(y,x) being the regular solution of the Helmholtz equation with respect to the variable *y*, including the case y=x.

Hence (3) becomes:

(8) $$V\left(x\right)=\int_{\partial \Omega }L\left(y,x;\lambda \right)V\left(y\right)ds_{y},\;x\in \Omega ,$$

$$L\left(y,x;\lambda \right)=\left(E\left(\Psi \left(y,x;\lambda \right)v^{0}\right)D^{*}\left(\partial_{x}\right)\right)D\left(t^{T}\right).$$

Formula (8) can be generalized for the case when Ω is unbounded.

Suppose Ω lies inside a strip of the smallest width defined by:

$$0<y_{2}<h,\;h=\frac{\pi }{\rho },\;\rho >0,$$

and ∂Ω extends to infinity.

So next we consider an unbounded domain $\Omega \subset R^{2}$ finitely connected, having a piecewise smooth boundary ∂Ω (∂Ω—extends to infinity).

Let $\Omega_{R}$ be the part of Ω situated inside a circle centered at zero, having radius *R*:

$$\Omega_{R}=\left\{y:y\in \Omega ,\;\left|y\right|<R\right\},\;\Omega_{R}^{\infty }=\Omega \backslash \Omega_{R},\;R>0.$$

**Theorem** **1.** *Consider V(y)∈A(Ω). If ∀x∈Ω,x fixed, we have*

(9) $$\lim_{R\to \;\infty }\int_{\Omega_{R}^{\infty }}L\left(y,x;\lambda \right)V\left(y\right)ds_{y}=0,$$

*then the Formula (8) is true.*

**Proof.**  For $x\in \Omega \;\;(\left|x\right|<R),x$ fixed, using (8) into account, we get

$$\begin{array}{c} \int_{\partial \Omega }L\left(y,x;\lambda \right)V\left(y\right)ds_{y}=\int_{\partial \Omega_{R}}L\left(y,x;\lambda \right)V\left(y\right)ds_{y}\\ +\int_{\partial \Omega_{R}^{\infty }}L\left(y,x;\lambda \right)V\left(y\right)ds_{y}=V\left(x\right)+\int_{\partial \Omega_{R}^{\infty }}L\left(y,x;\lambda \right)V\left(y\right)ds_{y},\;x\in \Omega_{R}. \end{array}$$

Taking into account condition (9), for R→∞, we obtain (8).

We also suppose

(10) $$\int_{\partial \Omega }\exp\left[-d_{0}\rho_{0}\left|y_{1}\right|\right]\;ds_{y}<\infty ,\;0<\rho_{0}<\rho ,$$

for some $d_{0}>0,$ and

(11) $$\left|V(y)\right|\leq \exp\left[\exp\rho_{2}\left|y_{1}\right|\right],\;\rho_{2}<\rho ,\;y\in \Omega .$$

In (6) we put

(12) $$\begin{array}{c} K\left(z\right)=\exp\left[-d\;\;i\rho_{1}\left(z-\frac{h}{2}\right)-d_{1}\;i\rho_{0}\left(d-\frac{h}{2}\right)\right],\\ K\left(x_{2}\right)=\exp\left[d\;\cos\rho_{1}\left(x_{2}-\frac{h}{2}\right)+d_{1}\;\cos i\rho_{0}\left(x_{2}-\frac{h}{2}\right)\right],\\ 0<\rho_{1}<\rho ,\;0<x_{2}<h, \end{array}$$

where

$$d=2ce^{\rho_{1}\left|x_{1}\right|},\;d_{1}>\frac{d_{0}}{\cos\left(\rho_{0}\frac{h}{2}\right)},\;c\geq 0,\;d>0.$$

Hence (8) holds.

Consider x∈Ω be fixed and y→∞. In the following we estimate the function Ψ(y,x;λ) and also its derivatives $\frac{\partial \Psi (y,x;\lambda )}{\partial y_{j}},\;j\in \left\{1,2\right\}$. For the estimation $\frac{\partial \Psi (y,x;\lambda )}{\partial y_{j}}$ we use equalities

(13) −2πK(x2)∂Ψ(y,x;λ)∂y1=(y1−x1)ReK(z0)−sign(y1−x1)(y2−x2)ImK(z0)r2−−(y1−x1)λ∫0∞a2+α2ReK(w)−(y2−x2)ImK(z)a2+r2·I1(λa)daa2+α2,y≠x,z0=iy1−x1+y2,I1(λa)=I0′(λa)

and

(14) −2πK(x2)∂Ψ(y,x;λ)∂y2=(y2−x2)ReK(z0)−(y1−x1)ImK(z0)r2−−λ∫0∞(y2−x2)ReK(z)−a2+α2ImK(z)a2+r2I1(λa)du,y1≠x1,

which are obtained from (6).

Really,

exp−diρ1z−h2−d1iρ0z−h2=expRe−diρ1z−h2−d1iρ0z−h2=exp−dρ1a2+α2cosρ1y2−h2−d1ρ0a2+α2cosρ0y2−h2.

As

$$\begin{array}{c} -\frac{\pi }{2}\leq -\frac{\rho_{1}}{\rho }\cdot \frac{\pi }{2}\leq \frac{\rho_{1}}{\rho }\cdot \frac{\pi }{2}<\frac{\pi }{2},\\ -\frac{\pi }{2}\leq -\frac{\rho_{1}}{\rho }\cdot \frac{\pi }{2}\leq \rho_{0}\left(y_{2}-\frac{h}{2}\right)\leq \frac{\rho_{1}}{\rho }\cdot \frac{\pi }{2}<\frac{\pi }{2}. \end{array}$$

Consequently,

$$\cos\rho \left(y_{2}-\frac{h}{2}\right)>0,\;\cos\rho_{0}\left(y_{2}-\frac{h}{2}\right)\geq \cos\frac{h\rho_{0}}{2}>\delta_{0}>0,$$

It does not vanish in the region Ω and

$$\begin{array}{c} \left|\Psi (y,x;\lambda )\right|=O\left[\exp\left(-\varepsilon \;\rho_{1}\left|y_{1}\right|\right)\right],\;\varepsilon >0,\;y\to \infty ,\;y\in \Omega ⋃\partial \Omega ,\\ \left|\frac{\partial \Psi (y,x;\lambda )}{\partial y_{j}}\right|=O\left[\exp\left(-\varepsilon \;\rho_{1}\left|y_{1}\right|\right)\right],\;\varepsilon >0,\;y\to \infty ,\;y\in \Omega ⋃\partial \Omega ,\;j\in \left\{1,2\right\}. \end{array}$$

We now choose $\rho_{1}$ with the condition $\rho_{2}<\rho_{1}<\rho$. Then condition (10) is fulfilled and the integral formula (8) is true. Theorem 1 is proved. □

Condition (12) may be weakened.

Consider

(15) $$A_{\rho }\left(\Omega \right)=\left\{V\left(y\right)\in A\left(\Omega \right),\;\left|V(y)\right|\leq \exp\left[O\left(\exp\rho \left|y_{1}\right|\right)\right],\;y\to \infty ,\;y\in \Omega \right\}.$$

**Theorem** **2.** *If $V\left(y\right)\in A_{\rho }\left(\Omega \right)$ satisfies the growth condition*

(16) $$\begin{array}{c} \left|V(y)\right|\leq C\exp\left[c\cos\rho_{1}\left(y_{2}-\frac{h}{2}\right)\exp\left(\rho_{1}\left|y_{1}\right|\right)\right],\\ c\geq 0,\;0<\rho_{1}<\rho ,\;y\in \partial \Omega , \end{array}$$

*C constant, then (8) is true.*

**Proof.**  Divide Ω by a line $y_{2}=\frac{h}{2}$ into the following two domains:

$$\Omega_{1}=\left\{y:\;0<y_{2}<\frac{h}{2}\right\}\;\mathrm{and}\;\Omega_{2}=\left\{y:\;\frac{h}{2}<y_{2}<h\right\}$$.

Consider first the domain $\Omega_{1}$. We put $K_{1}\left(z\right)$ in (6),

(17) $$\begin{array}{c} K_{1}\left(z\right)=K\left(z\right)\exp\left[-\delta \;i\tau \left(z-\frac{h}{2}\right)-\delta_{1}i\rho \left(z-\frac{h}{2}\right)\right],\\ \rho <\tau <2\rho ,\;\delta >0,\;\delta_{1}>o, \end{array}$$

K(z) being given by (12). With this notation, (10) is true.

Really,

$$\begin{array}{c} \left|\exp\left[-i\tau \left(z-\frac{h}{4}\right)-\delta_{1}i\rho \left(z-\frac{h}{4}\right)\right]\right|\\ =\exp\left[-\delta \;\tau \sqrt{a^{2}+\alpha^{2}}\cos\tau \left(y_{2}-\frac{h}{4}\right)\right]\\ =\exp\left[-\delta \;\tau \sqrt{a^{2}+\alpha^{2}}\right]\leq \exp\left[-\delta \exp\tau \left|y_{1}\right|\right], \end{array}$$

$$-\frac{\pi }{2}\leq -\tau \frac{\pi }{4}\leq \tau \left(y_{2}-\frac{h}{4}\right)\leq \tau \frac{\pi }{2}<\frac{h}{2}\;\mathrm{and}\;\cos\tau \left(y_{2}-\frac{h}{4}\right)\geq \cos\tau \frac{h}{4}\geq \delta_{0}>0$$.

Let denote by $\Psi^{+}\left(y,x;\lambda \right)$ the corresponding function Ψ(y,x;λ). As

$$\cos\tau \left(y_{2}-\frac{h}{4}\right)\geq \delta_{0},\;y\in \Omega_{1}⋃\partial \Omega_{1},$$

then for fixed $x\in \Omega_{1},\;y\in \Omega_{1}⋃\partial \Omega_{1}$,

$$\begin{array}{c} \left|\Psi^{+}\left(y,x;\lambda \right)\right|=O\left[\exp(-\delta_{0}\;\exp\left(\tau \left|y_{1}\right|\right)\right],\;y\to \infty ,\;\rho <\tau <2\rho ,\\ \left|\frac{\partial \Psi^{+}\left(y,x;\lambda \right)}{\partial y_{j}}\right|=O\left[\exp(-\delta_{0}\;\exp\left(\tau \left|y_{1}\right|\right)\right],\;y\to \infty ,\;\rho <\tau <2\rho ,\;j\in \left\{1,2\right\}. \end{array}$$

Suppose that $V\left(y\right)\in A_{\rho }\left(\Omega_{1}\right)$ satisfies:

(18) $$\left|V(y)\right|\leq C\exp\left[\exp\left(2\rho -\varepsilon \right)\left|y_{1}\right|\right],\;\varepsilon >0,\;\forall y\in \Omega_{1}.$$

We consider τ such that 2ρ−ε<τ<2ρ in (17).

Hence (17) is satisfied for the region $\Omega_{1}$, so

(19) $$V\left(x\right)=\int_{\partial \Omega_{1}}L\left(y,x;\lambda \right)V\left(y\right)ds_{y},\;x\in \Omega_{1},$$

$$L\left(y,x;\lambda \right)=\left(E\left(\Psi^{+}\left(y,x;\lambda \right)v^{0}\right)D^{*}\left(\partial_{x}\right)\right)D\left(t^{T}\right).$$

If $V\left(y\right)\in A_{\rho }\left(\Omega_{2}\right)$ satisfies the growth condition (16) in $\Omega_{2}$, and 2ρ−ε<τ<2ρ, then

(20) $$V\left(x\right)=\int_{\partial \Omega_{2}}L\left(y,x;\lambda \right)V\left(y\right)ds_{y},\;x\in \Omega_{2},$$

$$L\left(y,x;\lambda \right)=\left(E\left(\Psi^{-}\left(y,x;\lambda \right)v^{0}\right)D^{*}\left(\partial_{x}\right)\right)D\left(t^{T}\right).$$

Here $\Psi^{-}\left(y,x;\lambda \right)$ it is defined by the formula (6), in which K(z) it is replaced by the function $K_{2}\left(z\right):$

(21) $$K_{2}\left(z\right)=K\left(z\right)\exp\left[-\delta \;i\tau \left(z-h_{1}\right)-\delta_{1}i\rho \left(z-\frac{h}{2}\right)\right]$$

where

$$h_{1}=\frac{h}{2}+\frac{h}{4},\;\frac{h}{2}<y_{2}<h,\;\frac{h}{2}<x_{2}<h_{1},\;\delta >0,\;\delta_{1}>0.$$

In the formulas obtained with this formula, the integrals (according to (11)) converge uniformly for δ≥0, when $V\left(y\right)\in A_{\rho }\left(\Omega \right)$. In these formulas we put δ=0, hence

(22) $$V\left(x\right)=\int_{\partial \Omega }L\left(y,x;\lambda \right)V\left(y\right)ds_{y},\;x\in \Omega ,\;x_{2}\neq \frac{h}{2},$$

$$L\left(y,x;\lambda \right)=\left(E\left(\tilde{\Psi }\left(y,x;\lambda \right)v^{0}\right)D^{*}\left(\partial_{x}\right)\right)D\left(t^{T}\right).$$

(integrals over the cross section $y_{2}=\frac{h}{2}$ are mutually destroyed)

$$\tilde{\Psi }\left(y,x;\lambda \right)={\left(\Psi^{+}\left(y,x;\lambda \right)\right)}_{\delta =0}={\left(\Psi^{-}\left(y,x;\lambda \right)\right)}_{\delta =0}.$$

$\tilde{\Psi }\left(y,x;\lambda \right)$ is obtained here by (6), K(z) being given by (17), where δ=0 is considered. Using now the continuation principle, (22) holds, ∀x∈Ω. Under condition (18) and (22) holds, $\forall \delta_{1}\geq 0$. Considering $\delta_{1}=0$, Theorem 2 is proved. □

Choosing

(23) $$\begin{array}{c} K\left(z\right)=\frac{1}{z-x_{2}+2h}\exp\left(\sigma z\right),\\ K\left(x_{2}\right)=\frac{1}{2h}\exp\left(\sigma x_{2}\right),\;0<x_{2}<h,\;h=\frac{\pi }{\rho }, \end{array}$$

in (6), we get

(24) Φσ(y,x)=−e−σx2π(h)−1∫0∞Imexp(σz)(z−x2+2h)(z−x2)aI0(λa)a2+α2da.

Hence (8) becomes:

(25) $$V\left(x\right)=\int_{\partial \Omega }L_{\sigma }\left(y,x;\lambda \right)V\left(y\right)ds_{y},\;x\in \Omega ,$$

$$L_{\sigma }\left(y,x;\lambda \right)=\left(E\left(\Psi_{\sigma }\left(y,x;\lambda \right)v^{0}\right)D^{*}\left(\partial_{x}\right)\right)D\left(t^{T}\right).$$

## 3. Regularized Solution of the Cauchy Problem

**Theorem** **3.** *Let $V\left(y\right)\in A_{\rho }\left(\Omega \right)$ satisfying*

(26) $$\left|V(y)\right|\leq M,\;y\in T.$$

*If*

(27) $$V_{\sigma }\left(x\right)=\int_{\Sigma }L_{\sigma }\left(y,\;x;\lambda \right)V\left(y\right)ds_{y},\;x\in \Omega ,$$

*then:*

(28) $$\left|V\left(x\right)-V_{\sigma }\left(x\right)\right|\leq K_{\rho }\left(\lambda ,x\right)\sigma Me^{-\sigma x_{2}},\;x\in \Omega ,$$

(29) $$\left|\frac{\partial V(x)}{\partial x_{j}}-\frac{\partial V_{\sigma }\left(x\right)}{\partial x_{j}}\right|\leq K_{\rho }\left(\lambda ,x\right)\sigma Me^{-\sigma x_{2}},\;\sigma >1,\;x\in \Omega ,\;j\in \left\{1,2\right\},$$

*where*$K_{\rho }\left(\lambda ,\;x\right)$*are bounded functions on compact subsets of the domain*Ω.

**Proof.**  We prove first (28). Using (25) and (27), we have

$$\begin{array}{c} V\left(x\right)=\int_{\Sigma }L_{\sigma }\left(y,\;x;\lambda \right)U\left(y\right)ds_{y}+\int_{T}L_{\sigma }\left(y,\;x;\lambda \right)V\left(y\right)ds_{y}\\ =L_{\sigma }\left(x\right)+\int_{T}L_{\sigma }\left(y,\;x;\lambda \right)V\left(y\right)ds_{y},\;x\in \Omega . \end{array}$$

Using now (26), we obtain

(30) $$\begin{array}{c} \begin{array}{c} \left|V\left(x\right)-V_{\sigma }\left(x\right)\right|\leq \left|\int_{T}L_{\sigma }\left(y,\;x;\lambda \right)V\left(y\right)ds_{y}\right|\leq \end{array}\\ \leq \int_{T}\left|L_{\sigma }\left(y,\;x;\lambda \right)\right|\left|V(y)\right|ds_{y}\leq M\int_{T}\left|L_{\sigma }\left(y,\;x;\lambda \right)\right|ds_{y},\;x\in \Omega . \end{array}$$

We estimate now $\int_{T}\left|\Psi_{\sigma }\left(y,\;x;\lambda \right)\right|ds_{y}$ and $\int_{T}\left|\frac{\partial \Psi_{\sigma }\left(y,\;x;\lambda \right)}{\partial y_{j}}\right|ds_{y},\;j\in \left\{1,2\right\}$.

Using (24), we have

(31) $$\begin{array}{c} \Psi_{\sigma }\left(y,\;x\right)=\frac{e^{\sigma (y_{2}-x_{2})}}{\pi {\left(h\right)}^{-1}}\left[\int_{0}^{\infty }\left(\frac{\left(\beta +\beta_{1}\right)\cos\sigma \alpha_{1}}{\left(\alpha_{1}^{2}+\beta_{1}^{2}\right)\left(\alpha_{1}^{2}+\beta^{2}\right)}\right.\right.\\ +\left.\left.\frac{\left(-\alpha_{1}^{2}+\beta_{1}\beta \right)}{\left(\alpha_{1}^{2}+\beta_{1}^{2}\right)\left(\alpha_{1}^{2}+\beta^{2}\right)}\frac{\sin\sigma \alpha_{1}}{\alpha_{1}}\right)a\;I_{0}\left(\lambda a\right)\;da\right], \end{array}$$

where

$$\alpha_{1}^{2}=a^{2}+\alpha^{2},\;\beta =y_{2}-x_{2},\;\beta_{1}=y_{2}-x_{2}+2h.$$

Given (31) and the inequality

(32) $$I_{0}\left(\lambda a\right)\leq \sqrt{\frac{2}{\lambda \pi a}},$$

we have

(33) $$\int_{T}\left|\Psi_{\sigma }\left(y,\;x;\lambda \right)\right|ds_{y}\leq K_{\rho }\left(\lambda ,x\right)\sigma e^{-\sigma x_{2}},\;\sigma >1,\;x\in \Omega .$$

Using now

(34) $$\begin{array}{c} \frac{\partial \Psi_{\sigma }\left(y,\;x;\lambda \right)}{\partial y_{j}}=\frac{\partial \Psi_{\sigma }\left(y,\;x;\lambda \right)}{\partial s}\frac{\partial s}{\partial y_{j}}=2\left(y_{j}-x_{j}\right)\frac{\partial \Psi_{\sigma }\left(y,\;x;\lambda \right)}{\partial s},\\ s=\alpha^{2},\;j\in \left\{1,2\right\}, \end{array}$$

according to (31) and (32) we get

(35) $$\int_{T}\left|\frac{\partial \Psi_{\sigma }\left(y,\;x;\lambda \right)}{\partial y_{1}}\right|ds_{y}\leq K_{\rho }\left(\lambda ,x\right)\sigma e^{-\sigma x_{2}},\;\sigma >1,\;x\in \Omega ,$$

According to (31) and (32), we have

(36) $$\int_{T}\left|\frac{\partial \Psi_{\sigma }\left(y,\;x;\lambda \right)}{\partial y_{2}}\right|ds_{y}\leq K_{\rho }\left(\lambda ,x\right)\sigma e^{-\sigma x_{2}},\;\sigma >1,\;x\in \Omega ,$$

Using the inequalities (33), (35), (36) and (30), we get the estimate (28).

We prove now (29). From (25) and (27) we get:

(37) $$\begin{array}{c} \frac{\partial V(x)}{\partial x_{j}}=\int_{\Sigma }\frac{\partial L_{\sigma }\left(y,\;x;\lambda \right)}{\partial x_{j}}V\left(y\right)ds_{y}+\int_{T}\frac{\partial L_{\sigma }\left(y,\;x;\lambda \right)}{\partial x_{j}}V\left(y\right)ds_{y},\\ \frac{\partial V_{\sigma }\left(x\right)}{\partial x_{j}}=\int_{\Sigma }\frac{\partial L_{\sigma }\left(y,\;x;\lambda \right)}{\partial x_{j}}V\left(y\right)ds_{y},\;x\in \Omega ,\;j\in \left\{1,2\right\}. \end{array}$$

According to (37) and (26), we have

(38) $$\begin{array}{c} \begin{array}{c} \left|\frac{\partial V(x)}{\partial x_{j}}-\frac{\partial_{\sigma }V\left(x\right)}{\partial x_{j}}\right|\leq \left|\int_{T}\frac{\partial L_{\sigma }\left(y,\;x;\lambda \right)}{\partial x_{j}}V\left(y\right)ds_{y}\right| \end{array}\\ \leq \int_{T}\left|\frac{\partial L_{\sigma }\left(y,\;x;\lambda \right)}{\partial x_{j}}\right|\left|V(y)\right|ds_{y}\leq M\int_{T}\left|\frac{\partial L_{\sigma }\left(y,\;x;\lambda \right)}{\partial x_{j}}\right|ds_{y},\\ x\in \Omega ,\;j\in \{1,2\}. \end{array}$$

We estimate now $\int_{T}\left|\frac{\partial \Psi_{\sigma }\left(y,\;x;\lambda \right)}{\partial x_{1}}\right|ds_{y}$ and $\int_{T}\left|\frac{\partial \Psi_{\sigma }\left(y,\;x;\lambda \right)}{\partial x_{2}}\right|ds_{y}$ on the part *T* of the plane $y_{2}=0$.

We use

(39) $$\begin{array}{c} \frac{\partial \Psi_{\sigma }\left(y,\;x;\lambda \right)}{\partial x_{1}}=\frac{\partial \Psi_{\sigma }\left(y,\;x;\lambda \right)}{\partial s}\frac{\partial s}{\partial x_{1}}=-2\left(y_{1}-x_{1}\right)\frac{\partial \Psi_{\sigma }\left(y,\;x;\lambda \right)}{\partial s},\\ s=\alpha^{2}, \end{array}$$

for the estimation of the first integral.

From (31) and (32) and (39), we have

(40) $$\int_{T}\left|\frac{\partial \Psi_{\sigma }\left(y,\;x;\lambda \right)}{\partial x_{1}}\right|ds_{y}\leq K_{\rho }\left(\lambda ,x\right)\sigma e^{-\sigma x_{2}},\;\sigma >1,\;x\in \Omega .$$

According to (31) and (32), we have

(41) $$\int_{T}\left|\frac{\partial \Psi_{\sigma }\left(y,\;x;\lambda \right)}{\partial x_{2}}\right|ds_{y}\leq K_{\rho }\left(\lambda ,x\right)\sigma e^{-\sigma x_{2}},\;\sigma >1,\;x\in \Omega .$$

From inequalities (40), (41) and (38), we get (29). □

**Corollary** **1.** *We have*

$$\lim_{\sigma \to \infty }V_{\sigma }\left(x\right)=V\left(x\right),\;\lim_{\sigma \to \infty }\frac{\partial V_{\sigma }\left(x\right)}{\partial x_{j}}=\frac{\partial V(x)}{\partial x_{j}},\;j\in \left\{1,2\right\},\;\forall x\in \Omega .$$

Let

$${\bar{\Omega }}_{\varepsilon }=\left\{\left(x_{1},x_{2}\right)\in \Omega ,\;q>x_{2}\geq \varepsilon ,\;q=\max_{T}\psi \left(x_{1}\right),\;0<\varepsilon <q\right\},$$

$\psi (x_{1})$ being a curve and ${\bar{\Omega }}_{\varepsilon }\subset \Omega$ a compact set.

**Corollary** **2.** *If $x\in {\bar{\Omega }}_{\varepsilon }$, then the families of functions $\left\{V_{\sigma }\left(x\right)\right\}$ and $\left\{\frac{\partial V_{\sigma }\left(x\right)}{\partial x_{j}}\right\}$ converge uniformly for σ→∞, i.e.,*

$$V_{\sigma }\left(x\right)⇉V\left(x\right),\;\frac{\partial V_{\sigma }\left(x\right)}{\partial x_{j}}⇉\frac{\partial V(x)}{\partial x_{j}},\;j\in \left\{1,2\right\}.$$

We specify that the set $E_{\varepsilon }=\Omega \backslash {\bar{\Omega }}_{\varepsilon }$ is as a layer boundary for this problem.

Consider now the boundary of the domain Ω being composed of a hyper plane $y_{2}=0$ and a smooth curve Σ extending to infinity and lying in the strip

$$0<y_{2}<h,\;h=\frac{\pi }{\rho },\;\rho >0.$$

We consider Σ given

$$y_{2}=\psi \left(y_{1}\right),\;-\infty <y_{1}<\infty ,$$

where $\psi (y_{1})$ satisfies the condition

$$\left|\psi^{\prime }\left(y_{1}\right)\right|\leq P<\infty ,\;P=const.$$

We consider

$$q=\max_{T}\psi \left(y_{1}\right),\;l=\max_{T}\sqrt{1+\psi^{\prime 2}\left(y_{1}\right)}.$$

**Theorem** **4.** *If $V\left(y\right)\in A_{\rho }\left(\Omega \right)$ satisfies (26), and on a smooth curve* Σ *satisfies*

(42) $$\left|V(y)\right|\leq \delta ,\;0<\delta <1,$$

*then*

(43) $$\left|V(x)\right|\leq K_{\rho }\left(\lambda ,x\right)\sigma M^{1-\frac{x_{2}}{q}}\delta^{\frac{x_{2}}{q}},\;\sigma >1,\;x\in \Omega .$$

(44) $$\begin{array}{c} \left|\frac{\partial V(x)}{\partial x_{j}}\right|\leq K_{\rho }\left(\lambda ,x\right)\sigma M^{1-\frac{x_{2}}{q}}\delta^{\frac{x_{2}}{q}},\;\sigma >1,\;x\in \Omega ,\\ j\in \{1,2\}. \end{array}$$

**Proof.**  We prove first (43). From (25), we obtain

(45) $$V\left(x\right)=\int_{\Sigma }L_{\sigma }\left(y,\;x;\lambda \right)V\left(y\right)ds_{y}+\int_{T}L_{\sigma }\left(y,\;x;\lambda \right))V\left(y\right)ds_{y},\;x\in \Omega ,$$

and hence

(46) $$\left|V(x)\right|\leq \left|\int_{\Sigma }L_{\sigma }\left(y,\;x;\lambda \right)V\left(y\right)ds_{y}\right|+\left|\int_{T}L_{\sigma }\left(y,\;x;\lambda \right)V\left(y\right)ds_{y}\right|,\;x\in \Omega .$$

From (42), we have

(47) $$\begin{array}{c} \begin{array}{c} \left|\int_{\Sigma }L_{\sigma }\left(y,\;x;\lambda \right)V\left(y\right)ds_{y}\right|\leq \int_{\Sigma }\left|L_{\sigma }\left(y,\;x;\lambda \right)\right|\left|V(y)\right|ds_{y} \end{array}\\ \leq \delta \int_{\Sigma }\left|L_{\sigma }\left(y,\;x;\lambda \right)\right|ds_{y},\;x\in \Omega . \end{array}$$

We estimate now $\int_{\Sigma }\left|\Psi_{\sigma }\left(y,\;x;\lambda \right)\right|ds_{y},\;\int_{\Sigma }\left|\frac{\partial \Psi_{\sigma }\left(y,\;x;\lambda \right)}{\partial y_{1}}\right|ds_{y}$ and $\int_{\Sigma }\left|\frac{\partial \Psi_{\sigma }\left(y,\;x;\lambda \right)}{\partial y_{2}}\right|ds_{y}$ on Σ.

Given equality (31) and (32), we have

(48) $$\int_{\Sigma }\left|\Psi_{\sigma }\left(y,\;x;\lambda \right)\right|ds_{y}\leq K_{\rho }\left(\lambda ,x\right)\sigma e^{\sigma (q-x_{2})},\;\sigma >1,\;x\in \Omega .$$

Using now (31), (32) and (34), we get

(49) $$\int_{\Sigma }\left|\frac{\partial \Psi_{\sigma }\left(y,\;x;\lambda \right)}{\partial y_{1}}\right|ds_{y}\leq K_{\rho }\left(\lambda ,x\right)\sigma e^{\sigma (q-x_{2})},\;\sigma >1,\;x\in \Omega .$$

From (31) and (32), we have

(50) $$\int_{\Sigma }\left|\frac{\partial \Psi_{\sigma }\left(y,\;x;\lambda \right)}{\partial y_{2}}\right|ds_{y}\leq K_{\rho }\left(\lambda ,x\right)\sigma e^{\sigma (q-x_{2})},\;\sigma >1,\;x\in \Omega .$$

From (48)–(50) and applying (49), we get

(51) $$\left|\int_{\Sigma }L_{\sigma }\left(y,\;x;\lambda \right)V\left(y\right)ds_{y}\right|\leq K_{\rho }\left(\lambda ,x\right)\sigma \delta \;e^{\sigma (q-x_{2})},\;\sigma >1,\;x\in \Omega .$$

We know that

(52) $$\left|\int_{T}L_{\sigma }\left(y,\;x;\lambda \right)V\left(y\right)ds_{y}\right|\leq K_{\rho }\left(\lambda ,x\right)\sigma Me^{-\sigma x_{2}},\;\sigma >1,\;x\in \Omega .$$

According to (51), (52) and (46), we obtain

(53) $$\left|V(x)\right|\leq \frac{K_{\rho }\left(\lambda ,x\right)\sigma }{2}\left(\delta \;e^{\sigma q}+M\right)e^{-\sigma x_{2}},\;\sigma >1,\;x\in \Omega .$$

Considering

(54) $$\sigma =\frac{1}{q}\ln\frac{M}{\delta },$$

we get (43).

We prove now (44). From (25) we get:

(55) $$\begin{array}{c} \frac{\partial V(x)}{\partial x_{j}}=\int_{\Sigma }\frac{\partial L_{\sigma }\left(y,\;x;\lambda \right)}{\partial x_{j}}V\left(y\right)ds_{y}+\int_{T}\frac{\partial L_{\sigma }\left(y,\;x;\lambda \right)}{\partial x_{j}}V\left(y\right)ds_{y}\\ =\frac{\partial V_{\sigma }\left(x\right)}{\partial x_{j}}+\int_{T}\frac{\partial L_{\sigma }\left(y,\;x;\lambda \right)}{\partial x_{j}}V\left(y\right)ds_{y},\;x\in \Omega ,\;j\in \left\{1,2\right\}, \end{array}$$

where

(56) $$\frac{\partial V_{\sigma }\left(x\right)}{\partial x_{j}}=\int_{\Sigma }\frac{\partial L_{\sigma }\left(y,\;x;\lambda \right)}{\partial x_{j}}V\left(y\right)ds_{y}.$$

We get

(57) $$\begin{array}{c} \left|\frac{\partial V(x)}{\partial x_{j}}\right|\leq \left|\int_{\Sigma }\frac{\partial L_{\sigma }\left(y,x;\lambda \right)}{\partial x_{j}}V\left(y\right)ds_{y}\right|\\ \begin{array}{c} +\left|\int_{T}\frac{\partial L_{\sigma }\left(y,x;\lambda \right)}{\partial x_{j}}V\left(y\right)ds_{y}\right|\leq \left|\frac{\partial V_{\sigma }\left(x\right)}{\partial x_{j}}\right|\\ +\left|\int_{T}\frac{\partial L_{\sigma }\left(y,x;\lambda \right)}{\partial x_{j}}V\left(y\right)ds_{y}\right|,\;x\in \Omega ,\;j\in \left\{1,2\right\}. \end{array} \end{array}$$

From (42), we have:

(58) $$\begin{array}{c} \begin{array}{c} \left|\int_{\Sigma }\frac{\partial L_{\sigma }\left(y,\;x;\lambda \right)}{\partial x_{j}}V\left(y\right)ds_{y}\right|\leq \int_{\Sigma }\left|\frac{\partial L_{\sigma }\left(y,\;x;\lambda \right)}{\partial x_{j}}\right|\left|V(y)\right|ds_{y} \end{array}\\ \leq \delta \int_{\Sigma }\left|\frac{\partial L_{\sigma }\left(y,\;x;\lambda \right)}{\partial x_{j}}\right|ds_{y},\;x\in \Omega ,\;j\in \left\{1,2\right\}. \end{array}$$

Now we deal with $\int_{\Sigma }\left|\frac{\partial \Psi_{\sigma }\left(y,\;x;\lambda \right)}{\partial x_{1}}\right|ds_{y},$ and $\int_{\Sigma }\left|\frac{\partial \Psi_{\sigma }\left(y,\;x;\lambda \right)}{\partial x_{2}}\right|ds_{y}$ on Σ.

From (31), (32) and (39), we have

(59) $$\int_{\Sigma }\left|\frac{\partial \Psi_{\sigma }\left(y,\;x;\lambda \right)}{\partial x_{1}}\right|ds_{y}\leq K_{\rho }\left(\lambda ,x\right)\sigma e^{\sigma (q-x_{2})},\;\sigma >1,\;x\in \Omega ,$$

From (31) and (32), it follows:

(60) $$\int_{\Sigma }\left|\frac{\partial \Psi_{\sigma }\left(y,\;x;\lambda \right)}{\partial x_{2}}\right|ds_{y}\leq K_{\rho }\left(\lambda ,x\right)\sigma e^{\sigma (q-x_{2})},\;\sigma >1,\;x\in \Omega ,$$

From (59) and (60), bearing in mind (58), we have

(61) $$\begin{array}{c} \left|\int_{\Sigma }\frac{\partial L_{\sigma }\left(y,\;x;\lambda \right)}{\partial x_{j}}V\left(y\right)ds_{y}\right|\leq K_{\rho }\left(\lambda ,x\right)\sigma \delta e^{\sigma (q-x_{2})},\;\sigma >1,\;x\in \Omega ,\\ j\in \{1,2\}. \end{array}$$

We known that

(62) $$\begin{array}{c} \left|\int_{T}\frac{\partial L_{\sigma }\left(y,\;x;\lambda \right)}{\partial x_{j}}V\left(y\right)ds_{y}\right|\leq K_{\rho }\left(\lambda ,x\right)\sigma Me^{-\sigma x_{2}},\;\sigma >1,\;x\in \Omega ,\\ j\in \{1,2\}. \end{array}$$

According to (61), (62) and (57), we obtain

(63) $$\begin{array}{c} \left|\frac{\partial V(x)}{\partial x_{j}}\right|\leq \frac{K_{\rho }\left(\lambda ,x\right)\sigma }{2}\left(\delta \;e^{\sigma q}+M\right)e^{-\sigma x_{2}},\;\sigma >1,\;x\in \Omega ,\\ j\in \{1,2\}. \end{array}$$

Considering σ as in (54) we obtain (44). □

Assume that V(y)∈A(Ω) and instead of V(y) on Σ its continuous approximations $f_{\delta }\left(y\right)$ are given, with error 0<δ<1. We have

(64) $$\max_{\Sigma }\left|V\left(y\right)-f_{\delta }\left(y\right)\right|\leq \delta .$$

We put

(65) $$V_{\sigma (\delta )}\left(x\right)=\int_{\Sigma }N_{\sigma }\left(y,\;x;\lambda \right)f_{\delta }\left(y\right)ds_{y},\;x\in \Omega .$$

**Theorem** **5.** *If V(y)∈A(Ω) satisfies (26) on the plane $y_{2}=0$, then*

(66) $$\left|V\left(x\right)-V_{\sigma (\delta )}\left(x\right)\right|\leq K_{\rho }\left(\lambda ,x\right)\sigma M^{1-\frac{x_{2}}{q}}\delta^{\frac{x_{2}}{q}},\;\sigma >1,\;x\in \Omega ,$$

(67) $$\begin{array}{c} \left|\frac{\partial V(x)}{\partial x_{j}}-\frac{\partial V_{\sigma (\delta )}\left(x\right)}{\partial x_{j}}\right|\leq K_{\rho }\left(\lambda ,x\right)\sigma M^{1-\frac{x_{2}}{q}}\delta^{\frac{x_{2}}{q}},\;\sigma >1,\;x\in \Omega .\\ j\in \{1,2\}. \end{array}$$

**Proof.**  From (25) and (65), we get

$$\begin{array}{cc} \; & V\left(x\right)-V_{\sigma (\delta )}\left(x\right)=\int_{\partial \Omega }L_{\sigma }\left(y,x;\lambda \right)L\left(y\right)ds_{y}\\ \; & -\int_{\Sigma }L_{\sigma }\left(y,x;\lambda \right)f_{\delta }\left(y\right)ds_{y}=\int_{\Sigma }L_{\sigma }\left(y,x;\lambda \right)V\left(y\right)ds_{y}\\ \; & +\int_{T}L_{\sigma }\left(y,x;\lambda \right)V\left(y\right)ds_{y}-\int_{\Sigma }L_{\sigma }\left(y,x;\lambda \right)f_{\delta }\left(y\right)ds_{y}\\ \; & =\int_{\Sigma }L_{\sigma }\left(y,x;\lambda \right)\left\{V\left(y\right)-f_{\delta }\left(y\right)\right\}ds_{y}+\int_{T}L_{\sigma }\left(y,x;\lambda \right)L\left(y\right)ds_{y}. \end{array}$$

and

$$\begin{array}{cc} \; & \frac{\partial V(x)}{\partial x_{j}}-\frac{\partial V_{\sigma (\delta )}\left(x\right)}{\partial x_{j}}=\int_{\partial \Omega }\frac{\partial L_{\sigma }\left(y,x;\lambda \right)}{\partial x_{j}}V\left(y\right)ds_{y}\\ \; & -\int_{\Sigma }\frac{\partial L_{\sigma }\left(y,x;\lambda \right)}{\partial x_{j}}f_{\delta }\left(y\right)ds_{y}=\int_{\Sigma }\frac{\partial L_{\sigma }\left(y,x;\lambda \right)}{\partial x_{j}}V\left(y\right)ds_{y}\\ \; & +\int_{T}\frac{\partial L_{\sigma }\left(y,x;\lambda \right)}{\partial x_{j}}V\left(y\right)ds_{y}-\int_{\Sigma }\frac{\partial L_{\sigma }\left(y,x;\lambda \right)}{\partial x_{j}}f_{\delta }\left(y\right)ds_{y}\\ \; & =\int_{\Sigma }\frac{\partial L_{\sigma }\left(y,x;\lambda \right)}{\partial x_{j}}\left\{V\left(y\right)-f_{\delta }\left(y\right)\right\}ds_{y}+\int_{T}\frac{\partial L_{\sigma }\left(y,x;\lambda \right)}{\partial x_{j}}V\left(y\right)ds_{y},\\ \; & j\in \{1,2\}. \end{array}$$

From (26) and (64), we obtain:

$$\begin{array}{c} \left|V\left(x\right)-V_{\sigma (\delta )}\left(x\right)\right|=\left|\int_{\Sigma }L_{\sigma }\left(y,x;\lambda \right)\left\{V\left(y\right)-f_{\delta }\left(y\right)\right\}ds_{y}\right|\\ +\left|\int_{T}L_{\sigma }\left(y,x;\lambda \right)V\left(y\right)ds_{y}\right|\leq \int_{\Sigma }\left|L_{\sigma }\left(y,x;\lambda \right)\right|\left|\left\{V\left(y\right)-f_{\delta }\left(y\right)\right\}\right|ds_{y}\\ \begin{array}{c} +\int_{T}\left|L_{\sigma }\left(y,x;\lambda \right)\right|\left|V(y)\right|ds_{y}\leq \delta \int_{\Sigma }\left|L_{\sigma }\left(y,x;\lambda \right)\right|ds_{y}\\ +M\int_{T}\left|L_{\sigma }\left(y,x;\lambda \right)\right|ds_{y}. \end{array} \end{array}$$

and

$$\begin{array}{c} \left|\frac{\partial V(x)}{\partial x_{j}}-\frac{\partial V_{\sigma (\delta )}\left(x\right)}{\partial x_{j}}\right|=\left|\int_{\Sigma }\frac{\partial L_{\sigma }\left(y,x;\lambda \right)}{\partial x_{j}}\left\{V\left(y\right)-f_{\delta }\left(y\right)\right\}ds_{y}\right|\\ +\left|\int_{T}\frac{\partial L_{\sigma }\left(y,x;\lambda \right)}{\partial x_{j}}V\left(y\right)ds_{y}\right|\leq \int_{\Sigma }\left|\frac{\partial L_{\sigma }\left(y,x;\lambda \right)}{\partial x_{j}}\right|\left|\left\{U\left(y\right)-f_{\delta }\left(y\right)\right\}\right|ds_{y}\\ +\int_{T}\left|\frac{\partial L_{\sigma }\left(y,x;\lambda \right)}{\partial x_{j}}\right|\left|V(y)\right|ds_{y}\leq \delta \int_{\Sigma }\left|\frac{\partial L_{\sigma }\left(y,x;\lambda \right)}{\partial x_{j}}\right|ds_{y}\\ +M\int_{T}\left|\frac{\partial L_{\sigma }\left(y,x;\lambda \right)}{\partial x_{j}}\right|ds_{y},\;j\in \left\{1,2\right\}. \end{array}$$

Analog as in Theorems 3 and 4, we can prove that

$$\left|V\left(x\right)-V_{\sigma (\delta )}\left(x\right)\right|\leq \frac{K_{\rho }\left(\lambda ,x\right)\sigma }{2}\left(\delta \;e^{\sigma q}+M\right)e^{-\sigma x_{2}},$$

$$\left|\frac{\partial V(x)}{\partial x_{j}}-\frac{V_{\sigma (\delta )}\left(x\right)}{\partial x_{j}}\right|\leq \frac{K_{\rho }\left(\lambda ,x\right)\sigma }{2}\left(\delta \;e^{\sigma q}+M\right)e^{-\sigma x_{2}},\;j\in \left\{1,2\right\}.$$

Considering σ as in (54), we get (66) and (67). □

**Corollary** **3.** *We have*

$$\lim_{\delta \to 0}V_{\sigma (\delta )}\left(x\right)=V\left(x\right),\;\lim_{\delta \to 0}\frac{\partial V_{\sigma (\delta )}\left(x\right)}{\partial x_{j}}=\frac{\partial V(x)}{\partial x_{j}},\;j\in \left\{1,2\right\},\;\forall x\in \Omega .$$

**Corollary** **4.** *If $x\in {\bar{\Omega }}_{\varepsilon }$, then the families of functions $\left\{V_{\sigma (\delta )}\left(x\right)\right\}$ and $\left\{\frac{\partial V_{\sigma (\delta )}\left(x\right)}{\partial x_{j}}\right\}$ are convergent uniformly, for δ→0, i.e.,*

$$V_{\sigma (\delta )}\left(x\right)⇉V\left(x\right),\;\frac{\partial V_{\sigma (\delta )}\left(x\right)}{\partial x_{j}}⇉\frac{\partial V(x)}{\partial x_{j}},\;j\in \left\{1,2\right\}.$$

The following example illustrates the possibility of incorrect formulation of the classical Cauchy problem for system (1).

**Example** **1.** *Prove that the Cauchy problem for the following systems of linear partial differential equations is ill-posed:*

$$\left\{\begin{array}{c} \;\;\partial_{x_{1}}V_{1}-\partial_{x_{2}}V_{2}=0,\;\;\;\;\;\;\;\;\;\;\;\;\;\;\;\;\;\;\;\;\;\;\;\;\;\;\;\\ \;\;\;\;\partial_{x_{2}}V_{1}+\partial_{x_{1}}V_{2}=0,\\ -\partial_{x_{1}}V_{3}+\partial_{x_{2}}V_{4}=0,\;\;\;\;\;\;\;\;\;\;\;\;\;\;\;\;\;\;\;\;\;\;\;\;\;\;\;\\ \;\;\;\;\partial_{x_{2}}V_{3}+\partial_{x_{1}}V_{4}=0. \end{array}\right.$$

*Solutions to this system will be sought in the form*

$$\begin{array}{c} V_{1}=U_{1}e^{i(\lambda x_{1}+\mu x_{2})},\;V_{2}=U_{2}e^{i(\lambda x_{1}+\mu x_{2})},\\ V_{3}=U_{3}e^{i(\lambda x_{1}+\mu x_{2})},\;V_{4}=U_{4}e^{i(\lambda x_{1}+\mu x_{2})}. \end{array}$$

*Substituting these into the system, we obtain*

$$\begin{array}{c} \lambda^{2}+\mu^{2}=0,\;U_{1}=\frac{\lambda }{\mu }U_{2},\\ \lambda^{2}+\mu^{2}=0,\;U_{3}=\frac{\lambda }{\mu }U_{4}. \end{array}$$

*We choose the following μ=n,λ=−in. Then*

$$\begin{array}{c} V_{1n}=U_{1n}e^{nx_{1}-inx_{2}},\;V_{2n}=-iU_{1n}e^{nx_{1}-inx_{2}},\\ V_{3n}=U_{3n}e^{i(\lambda x_{1}+\mu x_{2})},\;V_{4n}=-iU_{3n}e^{nx_{1}-inx_{2}}. \end{array}.$$

*Separating the real part, we find the solutions*

$$\begin{array}{c} V_{1n}=U_{1n}e^{nx_{1}}\cos nx_{2},\;V_{2n}=U_{1n}e^{nx_{1}}\sin nx_{2},\\ V_{3n}=U_{3n}e^{nx_{1}}\cos nx_{2},\;V_{4n}=U_{3n}e^{nx_{1}}\sin nx_{2}. \end{array}$$

*The constants $U_{1n}$ and $U_{3n}$ are given by the formula $U_{1n}=U_{3n}=e^{-\sqrt{n}}.$*

*Hence*

$$\begin{array}{c} V_{1n}=e^{-\sqrt{n}}e^{nx_{1}}\cos nx_{2},\;V_{2n}=e^{-\sqrt{n}}e^{nx_{1}}\sin nx_{2},\\ V_{3n}=e^{-\sqrt{n}}e^{nx_{1}}\cos nx_{2},\;V_{4n}=e^{-\sqrt{n}}e^{nx_{1}}\sin nx_{2}. \end{array}$$

*The solutions $\left(V_{1n},V_{2n}\right)$, $\left(V_{3n},V_{4n}\right)$ satisfy at $x_{1}=0$ the following initial data:*

$$\begin{array}{c} V_{1n}\left(0,x_{2}\right)=\varphi_{1n}\left(x\right)=e^{-\sqrt{n}}\cos nx_{2},\;V_{2n}\left(0,x_{2}\right)=\varphi_{2n}\left(x\right)=e^{-\sqrt{n}}\sin nx_{2},\\ V_{3n}\left(0,x_{2}\right)=\varphi_{3n}\left(x\right)=e^{-\sqrt{n}}\cos nx_{2},\;V_{4n}\left(0,x_{2}\right)=\varphi_{4n}\left(x\right)=e^{-\sqrt{n}}\sin nx_{2}. \end{array}$$

*At n→∞, these initial data tend to zero. Moreover, their derivatives $\varphi_{1n}^{\left(k\right)}\left(x\right),\;\varphi_{2n}^{\left(k\right)}\left(x\right)$, $\varphi_{3n}^{\left(k\right)}\left(x\right),\;\varphi_{4n}^{\left(k\right)}\left(x\right)$ of orders k=1,2,…,p tend to zero as n→∞ (here, p− is an arbitrary fixed natural number). Indeed,*

*$\left.\begin{array}{c} \varphi_{1n}\left(x\right)=\pm n^{k}e^{-\sqrt{n}}\cos nx_{2}\\ \varphi_{2n}\left(x\right)=\pm n^{k}e^{-\sqrt{n}}\sin nx_{2} \end{array}\right\}$, if k− is even,*

*$\left.\begin{array}{c} \varphi_{1n}\left(x\right)=\pm n^{k}e^{-\sqrt{n}}\sin nx_{2}\\ \varphi_{2n}\left(x\right)=\pm n^{k}e^{-\sqrt{n}}\cos nx_{2} \end{array}\right\}$, if k− is odd,*

*$\left.\begin{array}{c} \varphi_{3n}\left(x\right)=\pm n^{k}e^{-\sqrt{n}}\cos nx_{2}\\ \varphi_{4n}\left(x\right)=\pm n^{k}e^{-\sqrt{n}}\sin nx_{2} \end{array}\right\}$, if k− is even,*

*$\left.\begin{array}{c} \varphi_{3n}\left(x\right)=\pm n^{k}e^{-\sqrt{n}}\sin nx_{2}\\ \varphi_{4n}\left(x\right)=\pm n^{k}e^{-\sqrt{n}}\cos nx_{2} \end{array}\right\}$, if k− is odd.*

*On the other hand, $V_{1n}\left(x_{1},x_{2}\right),\;V_{2n}\left(x_{1},x_{2}\right),\;V_{3n}\left(x_{1},x_{2}\right),\;V_{4n}\left(x_{1},x_{2}\right)$ is unbounded for any $x_{1}$.*

*We see that no matter what norm we choose to estimate the value of the initial data, we will not be able to assert that the smallness of this norm implies the smallness of the solution (the solution is estimated here by the maximum of its modulus). As admissible norms for the initial data, we here admit the following norms:*

$$\begin{array}{c} {∥\varphi_{1}\left(x\right)∥}_{p}=\max_{0\leq k\leq p}\underset{x_{2}}{\sup}\left|\varphi_{1}^{\left(k\right)}\left(x\right)\right|,\\ {∥\varphi_{2}\left(x\right)∥}_{p}=\max_{0\leq k\leq p}\underset{x_{2}}{\sup}\left|\varphi_{2}^{\left(k\right)}\left(x\right)\right|,\\ {∥\varphi_{3}\left(x\right)∥}_{p}=\max_{0\leq k\leq p}\underset{x_{2}}{\sup}\left|\varphi_{3}^{\left(k\right)}\left(x\right)\right|,\\ {∥\varphi_{4}\left(x\right)∥}_{p}=\max_{0\leq k\leq p}\underset{x_{2}}{\sup}\left|\varphi_{4}^{\left(k\right)}\left(x\right)\right|. \end{array}$$

*That is, there is no continuous dependence on the initial data and, therefore, the problem is set incorrectly. Thus, this problem does not have stability properties and, therefore, is ill-posed. We have seen that the solution of the Cauchy problem for this system is unstable. If we narrow the class of solutions under consideration to a compact set, then the problem becomes conditionally well-posed. To estimate the conditional stability, we can apply the results of the above theorems.*

**Example** **2.** *Let a system of partial differential equations of first order of the form*

$$\left\{\begin{array}{c} \;\;\;\;\frac{\partial V_{1}}{\partial x_{1}}-\frac{\partial V_{2}}{\partial x_{2}}+iV_{4}=0,\;\;\;\;\;\;\;\;\;\;\;\;\;\;\;\;\;\;\;\;\;\;\;\;\;\;\;\\ \;\;\;\;\frac{\partial V_{1}}{\partial x_{2}}+\frac{\partial V_{2}}{\partial x_{1}}+iV_{3}=0,\\ -\frac{\partial V_{3}}{\partial x_{1}}+\frac{\partial V_{4}}{\partial x_{2}}-iV_{2}=0,\;\;\;\;\;\;\;\;\;\;\;\;\;\;\;\;\;\;\;\;\;\;\;\;\;\;\;\\ \;\;\;\;\frac{\partial V_{3}}{\partial x_{2}}+\frac{\partial V_{4}}{\partial x_{1}}+iV_{1}=0. \end{array}\right.$$

*Check that the following relation holds:*

(68) $$D^{*}\left(\xi^{T}\right)D\left(\xi^{T}\right)=E\left(\left({\left|\xi \right|}^{2}+\lambda^{2}\right)v^{0}\right),\;v^{0}=\left(1,\ldots ,1\right)\in R^{n}.$$

*Assuming $\frac{\partial }{\partial x_{1}}\to \xi_{1}$ and $\frac{\partial }{\partial x_{2}}\to \xi_{2}$, compose the following matrices*

$$D\left(\xi^{T}\right)=\left(\begin{array}{c} \;\;\;\;\;\xi_{1}\;\;\;\;\xi_{2}\;\;\;\;\;\;\;\;0\;\;\;\;\;\;i\\ -\xi_{2}\;\;\;\;\xi_{1}\;-i\;\;\;\;\;\;0\\ \;\;\;\;\;\;0\;\;\;\;\;\;\;i\;\;\;-\xi_{1}\;\;\;\xi_{2}\\ \;\;\;\;\;\;i\;\;\;\;\;\;\;0\;\;\;\;\;\;\;\;\;\;\xi_{2}\;\;\;\;\xi_{1} \end{array}\right),\;\;\;\;\;D^{*}\left(\xi^{T}\right)=\left(\begin{array}{c} \;\;\xi_{1}-\xi_{2}\;\;\;\;\;\;\;0\;\;-i\\ \;\;\xi_{2}\;\;\;\;\;\;\;\xi_{1}\;-i\;\;\;\;\;\;\;\;0\\ \;\;\;0\;\;\;\;\;\;\;\;\;i\;\;\;-\xi_{1}\;\;\;\;\;\xi_{2}\\ -i\;\;\;\;\;\;\;\;0\;\;\;\;\;\;\;\;\;\xi_{2}\;\;\;\;\;\;\xi_{1} \end{array}\right).$$

*The relation (68) is easily checked.*

## 4. Conclusions

We have explicitly determined a regularized solution of the Cauchy problem for the matrix factorization Helmholtz’s equation in an unbounded two-dimensional domain. We specify that the approximate values of V(x) and $\frac{\partial V(x)}{\partial x_{j}},\;x\in \Omega ,\;j\in \left\{1,2\right\}$ must be determined, for solving applicable problems.

We have built a vector-functions family $V\left(x,f_{\delta }\right)=V_{\sigma (\delta )}\left(x\right)$ and $\frac{\partial V(x,f_{\delta })}{\partial x_{j}}=\frac{\partial V_{\sigma (\delta )}\left(x\right)}{\partial x_{j}}$, (j∈{1,2}) depending on σ (which is a parameter) and we have proved that for certain choices of σ=σ(δ), δ→0, and under certain conditions, the family $V_{\sigma (\delta )}\left(x\right)$ and $\frac{\partial V_{\sigma (\delta )}\left(x\right)}{\partial x_{j}}$ converges to V(x) and respectively to $\frac{\partial V(x)}{\partial x_{j}},\;x\in \Omega$. Hence, $V_{\sigma (\delta )}\left(x\right)$ and $\frac{\partial V_{\sigma (\delta )}\left(x\right)}{\partial x_{j}}$ determine the regularization of the solution of problems (1) and (2).

## Data Availability

Not applicable.
